# Effect of chlorhexidine and urinary catheter infection prevention in a Brazilian coronary ICU

**DOI:** 10.1186/cc14160

**Published:** 2015-03-16

**Authors:** GM Plantier, CE Bosso, BN Azevedo, AC Correa, AL Silva, V Raso

**Affiliations:** 1 Instituto do Coração de Presidente Prudente, Brazil; 2 Faculdade de Medicina - UNOESTE, Presidente Prudente, Brazil; 3 Santa Casa de Presidente Prudente, Brazil

## Introduction

Urinary catheter insertion is a common procedure in ICUs and can be an important cause of infection in the hospital environment [1,2]. We aimed to analyze the effect of chlorhexidine on long-term urinary catheter insertion and urinary tract infection (UTI) during a 5-year period in patients admitted to a coronary ICU.

## Methods

Analysis of patients admitted to a coronary ICU of a medium-sized hospital in Brazil from January 2010 to May 2014. The institutional protocol of periprocedural antisepsis was changed from iodine-based antiseptic to chlorhexidine in 2012. The UTI diagnosis was based on urine culture (>10^5 ^colony-forming units per ml of urine) associated with at least one clinical/laboratory abnormality (fever >38°C, urination urgency, increased urinary frequency, dysuria, or suprapubic or lumbar pain). The UTI rate represents the urinary tract infections associated with long-term urinary catheter (patient with UTI associated with long-term urinary catheter divided by patients with long-term urinary catheter × 1,000).

## Results

The urinary tract infection rates were 4.8 (year 2010: patients·day^-1 ^(*n*: 2,511), long-term urinary catheter·day^-1 ^(*n*: 1,455), device usage rate 958%)), 4.4 (year 2011: patients·day^-1 ^(*n*: 2,529), long-term urinary catheter·day^-1 ^(*n*: 1,140), device usage rate (45%)), 0.0 (year 2012: patients·day^-1 ^(*n*: 2,660), long-term urinary catheter·day^-1 ^(*n*: 783), device usage rate (29%)), 0.0 (year 2013: patients·day^-1 ^(*n*: 2,573), long-term urinary catheter·day^-1 ^(*n*: 960), device usage rate (37%)), and 0.0 (year 2014: patients·day^-1 ^(*n*: 1,070), long-term urinary catheter·day^-1 ^(*n*: 444), device usage rate (42%)).

## Conclusion

The use of chlorhexidine in the periprocedural antisepsis of urinary catheterization contributed to the decrease of urinary tract infections associated with long-term urinary catheter in patients admitted to the coronary ICU.
